# Variation of the clavicle’s muscle insertion footprints – a cadaveric study

**DOI:** 10.1038/s41598-019-52845-8

**Published:** 2019-11-08

**Authors:** M. Herteleer, S. Vancleef, P. Herijgers, J. Duflou, I. Jonkers, J. Vander Sloten, S. Nijs

**Affiliations:** 1 Department of Traumatology, UZ Leuven, Herestraat 49, 3000 Leuven, Belgium; 2 Department of Mechanical Engineering, Biomechanical Engineering, Celestijnenlaan 300 – bus 2419, 3001 Leuven, Belgium; 3 Anatomy Skills Lab, Minderbroedersstraat 12 blok q – bus 1031, 3000 Leuven, Belgium; 4 Department of Mechanical Engineering, Celestijnenlaan 300 bus 2422, 3001 Leuven, Belgium; 5 Human Movement Biomechanics Research Group, Tervuursevest 101 bus 1501, 3001 Leuven, Belgium

**Keywords:** Anatomy, Anatomy, Skeletal muscle, Skeletal muscle

## Abstract

The muscle footprint anatomy of the clavicle is described in various anatomical textbooks but research on the footprint variation is rare. Our goal was to assess the variation and to create a probabilistic atlas of the muscle footprint anatomy. 14 right and left clavicles of anatomical specimens were dissected until only muscle fibers remained. 3D models with muscle footprints were made through CT scanning, laser scanning and photogrammetry. Then, for each side, the mean clavicle was calculated and non-rigidly registered to all other cadaveric bones. Muscle footprints were indicated on the mean left and right clavicle through the 1-to-1 mesh correspondence which is achieved by non-rigid registration. Lastly, 2 probabilistic atlases from the clavicle muscle footprints were generated. There was no statistical significant difference between the surface area (absolute and relative), of the originally dissected muscle footprints, of male and female, and left and right anatomical specimens. Visualization of all muscle footprints on the mean clavicle resulted in 72% (right) and 82% (left) coverage of the surface. The Muscle Insertion Footprint of each specimen covered on average 36.9% of the average right and 37.0% of the average left clavicle. The difference between surface coverage by all MIF and the mean surface coverage, shows that the MIF location varies strongly. From the probabilistic atlas we can conclude that no universal clavicle exists. Therefore, patient-specific clavicle fracture fixation plates should be considered to minimally interfere with the MIF. Therefore, patient-specific clavicle fracture fixation plates which minimally interfere with the footprints should be considered.

## Introduction

The anatomical description of muscle origins and insertions on the bone (footprint anatomy) is part of many basic anatomical textbooks such as Netter’s anatomy, Sobotta Atlas of anatomy and Gray’s Atlas of Anatomy. The muscles that attach to the clavicle are respectively the pectoral muscle, the deltoid muscle, the subclavian muscle, the trapezoid and the sternocleidomastoid muscle. Apart from the muscles there are also several ligamentous attachments between acromioclavicular joint on the lateral side and between the sternoclavicular joint on the medial side. The clavicle functions as a strut between the thorax and the upper limb so that the upper limb’s movement is not disturbed by being in contact with the thorax. Furthermore it allows the scapula to move on the thoracic wall. It also covers the thoracic outlet and the structures that lie within it^1–3^.

In previous anatomical studies the muscle footprints were described by manually measuring the position and surface of the muscle attachments using calipers^4–7^. With the use of coordinate measurement devices these footprints can be measured in 3 dimensions and give a more accurate measurement of the footprint surface^8–11^. Earlier studies, which focused on the osteological variation, demonstrated that there is a large heterogeneity in clavicle anatomy^12–15^. This heterogeneity can result in difficulties during fracture fixation as the currently used off-the-shelf anatomical plates rarely fit adequately^16–18^. However, to our knowledge, there are no studies that have focused on the anatomical variation of the muscle footprints regarding the clavicle. Insight in the muscle footprint variation, would be helpful in the design of better fitting plates as these muscle footprints can be disturbing when fixating the plate to the bone and detachment could lead to a compromised vascularization of the bone^19^. The goal of our study is to describe the anatomical variation of the muscle attachment sites and to develop a probabilistic atlas.

## Materials and Methods

### Reverse engineering workflow

All research was performed in accordance with guidelines and regulations of our institution (KU Leuven/UZ Leuven), and we confirm that informed consent was obtained from all participants who donated their body to the Anatomical Skills Lab (KU Leuven). After approval from the Ethical Committee (Commissie Medische Ethiek, UZ Leuven/KU Leuven) 14 right and 14 left clavicles were dissected from 14 phenol preserved cadavers (7 male and 7 female cadavers). In a first stage all gross fat tissue, ligamentous and soft tissue structures apart from muscle fibers were removed. In the second stage the sternoclavicular and acromioclavicular ligaments were cut, the clavicle was removed from the thorax and the dissection was meticulously continued until only muscle fibers and bone remained. The dissection was performed by the author MH and supervised and assessed by authors PH & SN.

**Figure 1:**
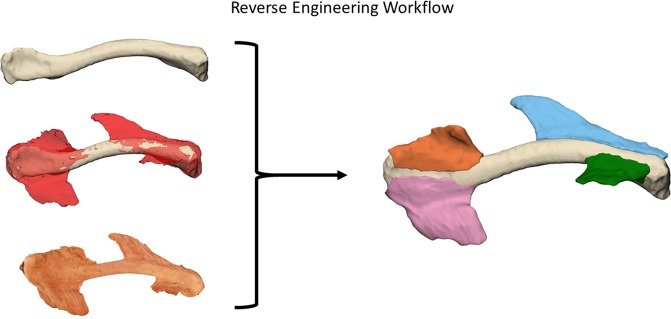
Reverse Engineering Workflow: The three different clavicle models: Top: Segmented CT scan model. Middle: Laser scanned muscle model (red) overlay on bone model (gray). Bottom: Photogrammetry model. These three models were used to reverse engineer each dissected clavicle in to a 3D model.

We created a workflow in order to reverse engineer the anatomical specimens in to usable 3D models, which is presented in Fig. 1. First, all clavicles were CT-scanned using a Siemens SOMATOM Definition Flash CT scanner with a 1 mm slice thickness and a resolution of 512 × 512 pixels. The CT images were then imported and segmented in Mimics Research 18.0 (Materialise, Leuven) to create 3D model bones. These STL files (3D mesh file format) were than remeshed using 3-matic 12.0 (Materialise, Leuven) to produce high quality STL files.

The dissected clavicles were then fixed in a frame in an upright position and optically scanned from 18 different angles using a Coord-3 CMM with LC60Dx Laser scanner (Nikon Metrology NV)^20^. The obtained point clouds of the surface model were meshed, optimized and exported as STL files using Focus Inspection v9.3 (Nikon Metrology NV).

Then, while remaining fixed in the frame, a large amount of photos were taken from different angles all around the clavicle (average of 192 photos per clavicle). The photos of each clavicle were imported into Agisoft photoscan (Agisoft, St Petersburg Russia) and exported as 3D photogrammetry PDF files (Adobe).

STL files of the soft tissue and bone were loaded into 3-matic (Materialise, Belgium) and aligned using the global registration module until an optimal alignment was obtained. Alignment of the bone with the soft tissue STL was judged by using the corresponding photogrammetry model. When necessary, small manual adjustments were made until optimal alignment was obtained.

The location of the attachment sites was obtained by subtracting the bone STL from the soft tissue STL. Based on the photogrammetry model, the STL model was manually improved until it represented the appearance of the photogrammetry model.

Basic measurements such as the surface of the muscle footprint and the surface and volume of the clavicle were performed.

**Figure 2:**
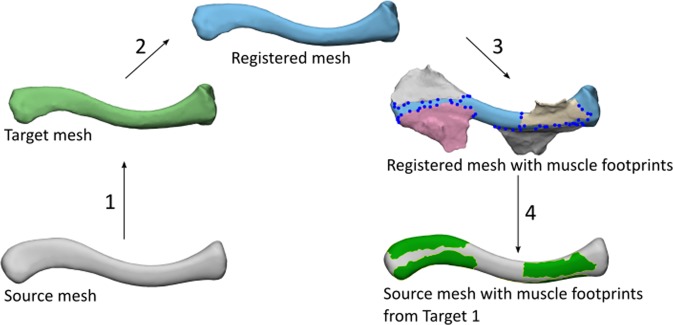
Non-Rigid Registration of the muscle attachment sites: First, the source (mean) mesh was transformed to the target mesh through a non-rigid registration method. The transformed mesh was then nearly identical to the target mesh (registered mesh). The muscle footprints of the target mesh were then indicated on the registered mesh.Through the one-to-one mesh correspondence between source and the registered meshes, muscle attachment sites in the registered mesh can also be highlighted in the mean mesh using vertex indices.. This was repeated for every 14 target meshes in order to create a muscle footprint atlas on the source (mean) mesh.

After creation of the STL files, one clavicle was non-rigidly registered to the other 13 clavicles using the algorithm based on Danckaers *et al*.^21^. The mean clavicle was calculated and then this clavicle (further referred to as ‘source’) was non-rigidly registered to all 14 left and 14 right cadaver clavicles respectively(further referred to as ‘target’)^22^. Next, the muscle footprints of each cadaver clavicle were copied to this corresponding registered mesh. Since the non-rigid registration algorithm assures one-on-one mesh correspondence between source and target, muscle footprints of the target can also be highlighted on the source by using the vertex indexes. This was repeated for all 14 clavicles and resulted in a surface footprint map. The framework of highlighting the muscle attachment sites is presented in Fig. 2.

The probabilistic area was constructed by averaging the attachment region by the number of muscles contributing to that particular attachment region. This procedure was performed for the left and right set of clavicles.

### Additional information

There are no competing financial or non-financial interests for any of the authors regarding the performed research presented in this manuscript.

## Results

The muscle footprint surfaces and the surface and volume of the clavicles are shown in Table 1. A mean coverage of 37.5% in right and 35.5% in left clavicles was observed in the original clavicles.

**Table 1:** Propereties of individual right clavicles (expressed as absolute and % values) and comparison of the means between male and female clavicles.

	Length (mm)	Volume (mm³)	Surface Bone (mm²)	Deltoid (mm²)	%	Trapezoid (mm²)	%	Pectoral (mm²)	(%)	Sternocleidomastoid (mm²)	%	Sublcavian (mm²)	%	Total % covered by muscle
Male	176	51.876	12.501	902	7	1.073	9	771	6	820	7	243	2	30
Male	141	32.684	8.577	513	6	1.231	14	837	10	469	5	838	10	45
Male	160	39.174	10.210	678	7	1.318	13	785	8	678	7	586	6	40
Male	140	36.935	9.461	723	8	1.228	13	781	8	582	6	455	5	40
Male	150	55.678	11.911	861	7	831	7	805	7	295	2	468	4	27
Male	158	37.192	10.133	729	7	928	9	716	7	521	5	358	4	32
Male	154	38.307	9.785	717	7	928	9	483	5	749	8	548	6	35
**MEAN**	**154**	**41**.**692**	**10**.**368**	**732**	**7**	**1**.**077**	**11**	**740**	**7**	**588**	**6**	**499**	**5**	**36**
SD	12	8.575	1.377	127	1	187	3	119	2	179	2	189	2	6
Female	160	32.148	9.001	589	7	873	10	848	9	664	7	466	5	38
Female	156	27.727	8.516	917	11	1.014	12	596	7	598	7	536	6	43
Female	151	29.484	8.434	631	7	934	11	621	7	195	2	512	6	34
Female	151	36.460	9.662	516	5	1.397	14	739	8	389	4	316	3	35
Female	151	26.063	8.048	510	6	1.118	14	525	7	663	8	496	6	41
Female	150	29.726	8.725	653	7	920	11	709	8	456	5	284	3	35
Female	122	28.488	7.562	782	10	707	9	953	13	579	8	621	8	48
**MEAN**	**149**	**30**.**014**	**8**.**564**	**657**	**8**	**995**	**12**	**713**	**8**	**506**	**6**	**462**	**5**	**39**
SD	12	3.407	672	147	2	218	2	149	2	171	2	121	2	5
T-test p-value	0,427	**0**,**010**	**0**,**013**	0,328	0,398	0,464	0,486	0,718	0,264	0,402	0,812	0,664	0,705	0,280

The mean absolute and relative surface areas of the respective male and female clavicle’s were compared using a paired t-test. There was a significant difference between the volume and surface area of the male and female clavicle’s and the footprint of the left deltoid muscle. (Tables 1 and 2). A paired t-test was also performed to detect significant differences in surface area between left and concomitant right muscle attachment sites but it did not reach statistical significance <0.05 for any of the muscle attachment sites. Detailed results of each muscle for each target can be found in the supplement table.

**Table 2:** Propereties of individual left clavicles (expressed as absolute and % values) and comparison of the means between male and female clavicles.

	Length (mm)	Volume (mm³)	Surface Bone (mm²)	Deltoid (mm²)	%	Trapezoid (mm²)	%	Pectoral (mm²)	(%)	Sternocleidomastoid (mm²)	%	Sublcavian (mm²)	%	Total % covered by muscle
Male	176	50.560	11.968	707	6	800	7	810	7	548	5	467	4	28
Male	148	34.364	9.083	866	10	976	11	1.131	12	563	6	634	7	46
Male	160	45.944	10.873	981	9	1.291	12	637	6	976	9	405	4	39
Male	148	37.228	9.574	480	5	1.614	17	935	10	708	7	137	1	40
Male	152	50.931	11.451	875	8	819	7	909	8	865	8	561	5	35
Male	161	34.442	9.687	698	7	972	10	375	4	444	5	415	4	30
Male	157	39.689	10.301	881	9	653	6	567	6	477	5	656	6	31
**MEAN**	**157**	**41**.**880**	**10**.**420**	**784**	**8**	**1**.**018**	**10**	**766**	**7**	**654**	**6**	**468**	**5**	**36**
SD	10	7.212	1.058	168	2	330	4	256	3	203	2	177	2	7
Female	160	31.471	8.947	449	5	1.023	11	588	7	186	2	278	3	28
Female	160	29.455	8.722	577	7	1.250	14	777	9	759	9	553	6	45
Female	150	28.983	8.496	636	7	713	8	380	4	309	4	365	4	28
Female	151	35.633	9.528	765	8	1.348	14	378	4	392	4	445	5	35
Female	148	21.867	7.213	656	9	746	10	691	10	432	6	361	5	40
Female	150	30.775	8.978	624	7	898	10	471	5	291	3	333	4	29
Female	121	26.063	7.167	406	6	987	14	383	5	746	10	553	8	43
**MEAN**	**149**	**29**.**178**	**8**.**436**	**588**	**7**	**995**	**12**	**524**	**6**	**445**	**5**	**413**	**5**	**35**
SD	13	4.335	907	124	1	239	2	164	2	224	3	108	2	7
T-test p-value	0,179	**0**,**003**	**0**,**003**	**0**,**030**	0,495	0,885	0,300	0,061	0,417	0,092	0,554	0,497	0,621	0,944

The mean area (±standard deviation) of the muscle attachment sites for the right and left clavicle on the source can be found in Table 3. The MIF of each specimen covered on average 36.9% of the average right and 37.0% of the average left clavicle.

**Table 3:** Mean surfaces +/− standard deviation of the left and right muscle footprints.

	Deltoid	Trapezoid	Pectoralis major	Sternocleidomastoid	Subclavian
	(mm^2^ ± SD)	(mm^2^ ± SD)	(mm^2^ ± SD)	(mm^2^ ± SD)	(mm^2^ ± SD)
Right	694 ± 133	1036 ± 192	726 ± 126	547 ± 167	481 ± 148
Left	686 ± 168	1006 ± 267	645 ± 233	550 ± 224	440 ± 138

**Figure 3:**
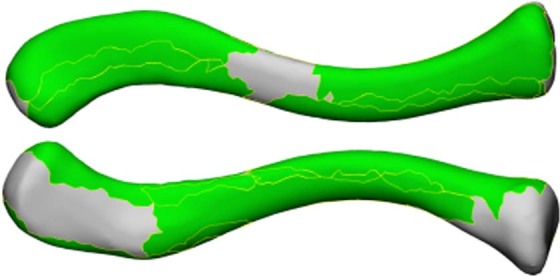
Visualization of the mean right clavicle with the union of all muscle footprints, resulting in a coverage of 72% of the clavicle. Top clavicle: superior view, Bottom clavicle: inferior view.

**Figure 4:**
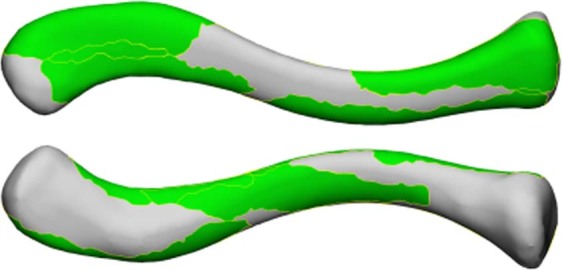
Visualization of the mean right clavicle covered by only the largest muscle footprints, resulting in a coverage of 52% of the clavicle. Top clavicle: superior view, Bottom clavicle: inferior view.

Visualization of all muscle footprints on the right average clavicle resulted in 72% coverage of the surface, visualizing only each muscle’s largest muscle footprint led to 52% coverage, which can be seen in Figs 3 and 4 respectively. All muscle footprints on the left average clavicle resulted in 82% coverage of the surface, each muscle’s largest muscle footprint led to 49% coverage.

**Figure 5:**
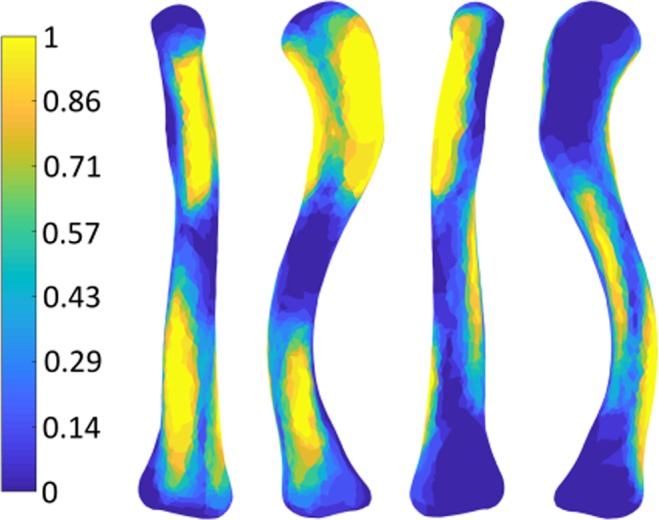
Probabilistic Atlas of the muscle attachment sites of the right clavicle: The bright yellow zones represent the areas where all 14 muscles overlap. The dark blue zones are the areas where no muscles overlap.

**Figure 6:**
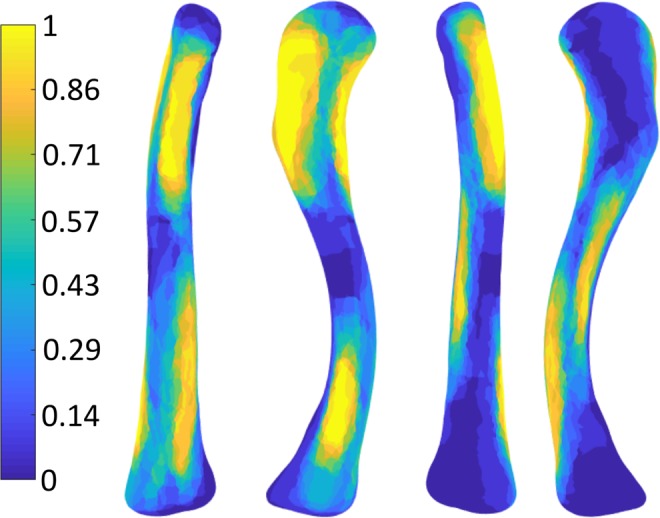
Probabilistic Atlas shown as Footprint Heatmap of the left clavicle (from left to right: anterior – superior – posterior – inferior view): The bright yellow zones represent the areas where all 14 muscles overlap. The dark blue zones are the areas where no muscles overlap.

In Fig. 5 the resulting probabilistic atlas of the right clavicle is shown, in Fig. 6 the resulting probabilistic atlas of the left clavicle is shown.

## Discussion

The goal of our study was to describe the variability of muscle attachment sites through the development of a probabilistic atlas of the muscle footprints on the clavicle. The large differences in muscle footprint areas, as shown by the standard deviation, already indicate their variability. The difference between coverage by all MIF and the average MIF area, shows that MIF location varies strongly as well.

Many previous studies described the bony anatomy of the clavicle and searched for constants in its highly variable anatomy in order to produce better fitting implants^13,14,22^. Simon Lambert *et al*. described an implant preferred pathway (IPP) which was defined as a continuous linear region where the least possible soft tissue disruption would be necessary for plate fixation^13^. The location of this IPP was based on expert opinion, defined on one clavicle and then registered through a non-rigid registration method to 174 clavicles. Fatah *et al*. described the bilateral direction asymmetry of clavicles using a statistical bone atlas^23^. They calculated a mean bone and used this to localize five muscle attachment sites based on a standard anatomical textbook. These sites were then propagated across their entire sample to generate correspondence between homologous anatomical sites. Our results support the existence of an implant preferred pathway as defined by Lambert *et al*. but there is an important remark to make. Lambert and Fatah both assumed that the muscle footprint and implant preferred pathway is not related to the shape of the bone and that a universal clavicle exists, which is in contradiction to our results, as no general IPP could be identified and a lot of variation is present in the muscle footprints on the mean clavicle. The creation and usage of the 3D muscle insertion footprint models in this study allowed the authors to exactly describe the location of the muscle on the bone. As the clavicle is a bone (or free shape from a mathematical/engineering point of view) with a very large shape variation, the position of a point/surface/muscle footprint cannot be easily described nor can it easily be extrapolated to another bone. The usage of a SSM and the according Non-Rigid-Registration allowed us to compare the position of the muscle insertion footprints on these highly variable shapes (clavicles) with each other. This reverse engineering technique requires the usage of high quality 3D models.

Fukuda *et al*. addressed the variation of muscle attachment regions for hip muscles^24^. In their study they used 8 cadaver specimens on which the muscle attachment regions were defined and a muscle probabilistic atlas was constructed. For each muscle in the probabilistic atlas, they created an average muscle footprint and used this to predict patient-specific muscle footprints. They suggested that the probabilistic atlas could be useful to estimate these patient-specific attachment regions. Prediction of the clavicle’s muscle footprint will be a topic of future research.

Two recent meta-analyses investigated the effect of anteroinferior versus superior plating of the clavicle and they both concluded that plating along the superior and anteroinferior aspects of the clavicle leads to similar operative outcomes with respect to union, nonunion, malunion, and implant failure^25,26^. Only the meta-analysis by Nourian *et al*. concluded that patients in the superior plating group had a significantly higher probability of suffering from symptomatic hardware as compared to patients in the anteroinferior plating group^25^. Baltes *et al*. looked in detail at the influence of plate positioning on hardware removal rate but could not conclude that it leads to reduced implant removal rates^27^. From an anatomical point of view our results support a superior plating position as this would lead to a less extensive muscle dissection. We do believe however that plate prominence is key to reduce soft tissue irritation. Galdi *et al*. compared the results of plates positioned anteroinferiorly of 2.7 mm vs 3.5 mm and concluded that the 2.7 mm plates resulted in excellent clinical outcomes but with a higher cosmetic applicability and a lower implant removal rate^28^.

Havet *et al*. described the periosteal vascularization of the middle one third of the clavicle^29^. They showed that main blood supply was periosteal and arose from two different origins. First, the posteroinferior surface of the clavicle is vascularized by the middle part of the suprascapular artery in all their cases. Secondly, in 75% of the cases the anterosuperior surface of the middle third was supplied by anastomosing vessels between the medial and lateral branches of the thoracocarcomial trunk. Because of this periosteal vascularization they conclude that there is a high risk of disruption of the periosteal blood supply in case of severely displaced fractures. These anatomical findings explain why in several randomized trials where operative vs conservative treatment of more than 2 cm displaced fractures resulted in non-union of the conservatively treated fractures^30,31^. Research has shown that muscle is capable of supplying osteoprogenitor cells in cases where the periosteum is insufficient, and the muscular osteoprogenitors possess similar osteogenic potential to those derived from the periosteum^32^. We believe that successful clavicle fracture treatment relies on anatomical preservation, meaning bony stability and soft tissue recovery in order to prevent non-union. Furthermore, disruption of muscular footprints accompanied by compromised perfusion contributes to the risk of complications^13^.

Goudie *et al*. tried described the relationship between clavicle shortening and functional outcome based on 3D-CT measurements^33^. They concluded that a reduced clinical outcome was predominantly mediated by the development of a non-union and see the prevention of non-union as the holy grail in successful clavicle fracture treatment. Although we agree with their findings, we believe that the reduced outcome is not only related to non-union but also to severe malunion which can lead to a change in preload of the muscles attaching to the clavicle and its long-term effect on arthrosis of the nearby glenohumeral joint^34,35^.

The strength of our study lies in its innovative character and the use of reverse engineering and non-rigid registration to describe the clavicle’s soft tissue anatomy. To our knowledge, this is the first study that describes the muscle footprint variation of the clavicle. It’s major weakness is the relatively small number of specimens used. This was due to the time consuming reverse engineering process and the technical pitfalls to standardize the process that came along with it. Although only a relatively small number of clavicles were used we were still able to demonstrate the large variability in muscle footprint anatomy.

In conclusion, our anatomical description encourages the use of fixation plates or intramedullary devices that respect the muscle footprints for the surgical treatment of displaced midshaft clavicle fractures. Soft tissue respecting fixation devices could lead to an even larger reduction of fracture non-union compared with conventional fixation devices and conservatively treated fractures. Since the probabilistic atlas showed the high variability of clavicle muscle footprints, patient-specific clavicle fixation plates that not only take the shape of the bone but also the position of the muscle footprints into account could potentially reduce surgery time, improve anatomical alignment, preserve muscle length and preload, and reduce the rate of non-union. We believe that our results create a basis for an improved approach in clavicle fracture research and optimizing the currently existing hardware.

## References

[1] Netter, F. *Atlas of Human Anatomy*. (Elsevier Health Sciences, 2014).

[2] Paulsen, F. *Sobotta Atlas of Human Anatomy*, *Vol*.*1*. (Elsevier Health Sciences, 2011).

[3] Standring, S. *Gray’s Anatomy The Anatomical Basis of Clinical Practice*. (Elsevier Health Sciences, 2015).

[4] Ruotolo C, Fow JE, Nottage WM (2004). The supraspinatus footprint: an anatomic study of the supraspinatus insertion. Arthrosc. J. Arthrosc. Relat. Surg..

[5] Carey P, Owens BD (2010). Insertional footprint anatomy of the pectoralis major tendon. Orthopedics.

[6] Feucht MJ (2015). Gross anatomical and dimensional characteristics of the proximal hamstring origin. Knee Surgery, Sport. Traumatol. Arthrosc..

[7] Obey MR, Broski SM, Spinner RJ, Collins MS, Krych AJ (2016). Anatomy of the Adductor Magnus Origin: Implications for Proximal Hamstring Injuries. Orthop. J. Sport. Med..

[8] Veeger HEJ, Van Der Helm FCT, Van Der Woude LHV, Pronk GM, Rozendal RH (1991). Inertia and muscle contraction parameters for musculoskeletal modelling of the shoulder mechanism. J. Biomech..

[9] Capo JT (2014). Three-dimensional analysis of elbow soft tissue footprints and anatomy. J. Shoulder Elbow Surg..

[10] Kamineni S, Bachoura A, Behrens W, Kamineni E, Deane A (2015). Distal Insertional Footprint of the Brachialis Muscle: 3D Morphometric Study. Anat. Res. Int..

[11] Yoo JC (2015). Subscapularis Tendon Tear Classification Based on 3-Dimensional Anatomic Footprint: A Cadaveric and Prospective Clinical Observational Study. Arthrosc. J. Arthrosc. Relat. Surg..

[12] Daruwalla ZJ, Courtis P, Fitzpatrick C, Fitzpatrick D, Mullett H (2010). Anatomic variation of the clavicle: A novel three-dimensional study. Clin. Anat..

[13] Lambert S (2016). Computerized tomography based 3D modelling of the clavicle. J. Orthop. Res..

[14] Andermahr J (2007). Anatomy of the clavicle and the intramedullary nailing of midclavicular fractures. Clin. Anat..

[15] Lu Y-C, Untaroiu CD (2013). Statistical shape analysis of clavicular cortical bone with applications to the development of mean and boundary shape models. Comput. Methods Programs Biomed..

[16] Malhas AM, Skarparis YG, Sripada S, Soames RW, Jariwala AC (2016). How well do contoured superior midshaft clavicle plates fit the clavicle? A cadaveric study. J. Shoulder Elb. Surg..

[17] Van Tongel A (2014). Evaluation of prominence of straight plates and precontoured clavicle plates using automated plate-to-bone alignment. Acta Orthop. Belg..

[18] Huang JI, Toogood P, Chen MR, Wilber JH, Cooperman DR (2007). Clavicular anatomy and the applicability of precontoured plates. J. Bone Joint Surg. Am..

[19] Baumgaertel F, Buhl M, Rahn BA (1998). Fracture healing in biological plate osteosynthesis. Injury.

[20] Metrology Nikon. LC60Dx. at, https://www.nikonmetrology.com/en-us/product/lc60dx.

[21] Danckaers, F. *et al*. Correspondence Preserving Elastic Surface Registration with Shape Model Prior. In *2014 22nd Int*. *Conf*. *Pattern Recognit*. 2143–2148, 10.1109/ICPR.2014.373 (IEEE, 2014).

[22] Vancleef, S. *et al*. Why off-the-shelf clavicle plates rarely fit: anatomic analysis of the clavicle through statistical shape modeling. *J*. *Shoulder Elb*. *Surg*, 10.1016/j.jse.2018.09.018 (2019). 10.1016/j.jse.2018.09.018 30609957

[23] Abdel Fatah EE (2012). A three-dimensional analysis of bilateral directional asymmetry in the human clavicle. Am. J. Phys. Anthropol..

[24] Fukuda N (2017). Estimation of attachment regions of hip muscles in CT image using muscle attachment probabilistic atlas constructed from measurements in eight cadavers. Int. J. Comput. Assist. Radiol. Surg..

[25] Nourian A, Dhaliwal S, Vangala S, Vezeridis PS (2017). Midshaft Fractures of the Clavicle: A Meta-analysis Comparing Surgical Fixation Using Anteroinferior Plating Versus Superior Plating. in. J. Orthop. Trauma.

[26] Ai J (2017). Anterior inferior plating versus superior plating for clavicle fracture: A meta-analysis. BMC Musculoskelet. Disord..

[27] Baltes TPA, Donders JCE, Kloen P (2017). What is the hardware removal rate after anteroinferior plating of the clavicle? A retrospective cohort study. J. Shoulder Elb. Surg..

[28] Galdi B (2013). Anteroinferior 2.7-mm versus 3.5-mm plating for AO/OTA type B clavicle fractures: a comparative cohort clinical outcomes study. J. Orthop. Trauma.

[29] Havet E (2008). Vascular anatomical basis of clavicular non-union. Surg. Radiol. Anat..

[30] Woltz S, Krijnen P, Schipper IB (2017). Plate Fixation Versus Nonoperative Treatment for Displaced Midshaft Clavicular Fractures: A Meta-Analysis of Randomized Controlled Trials. J. Bone Joint Surg. Am..

[31] McKee RC, Whelan DB, Schemitsch EH, McKee MD (2012). Operative versus nonoperative care of displaced midshaft clavicular fractures: a meta-analysis of randomized clinical trials. J. Bone Joint Surg. Am..

[32] Davis KM (2015). Muscle-bone interactions during fracture healing. J. Musculoskelet. Neuronal Interact..

[33] Goudie EB (2017). The Influence of Shortening on Clinical Outcome in Healed Displaced Midshaft Clavicular Fractures after Nonoperative Treatment. J. Bone Jt. Surg. - Am. Vol..

[34] Hillen RJ, Bolsterlee B, Veeger DHEJ (2016). The biomechanical effect of clavicular shortening on shoulder muscle function, a simulation study. Clin. Biomech. (Bristol, Avon).

[35] Weinberg DS, Vallier HA, Gaumer GA, Cooperman DR, Liu RW (2016). Clavicle Fractures are Associated With Arthritis of the Glenohumeral Joint in a Large Osteological Collection. J. Orthop. Trauma.

